# Emotional Content Modulates Attentional Visual Orientation During Free Viewing of Natural Images

**DOI:** 10.3389/fnhum.2018.00459

**Published:** 2018-11-15

**Authors:** Carolina Astudillo, Kristofher Muñoz, Pedro E. Maldonado

**Affiliations:** ^1^Biomedical Neuroscience Institute, Universidad de Chile, Santiago, Chile; ^2^Department of Neuroscience, Faculty of Medicine, Universidad de Chile, Santiago, Chile

**Keywords:** pupil, emotional processing, natural images, natural vision, attention

## Abstract

Visual attention is the process that enables us to select relevant visual stimuli in our environment to achieve a goal or perform adaptive behaviors. In this process, bottom-up mechanisms interact with top-down mechanisms underlying the automatic and voluntary orienting of attention. Cognitive functions, such as emotional processing, can influence visual attention by increasing or decreasing the resources destined for processing stimuli. The relationship between attention and emotion has been explored mainly in the field of automatic attentional capturing; especially, emotional stimuli are suddenly presented and detection rates or reaction times are recorded. Unlike these paradigms, natural visual scenes may be comprised in multiple stimuli with different emotional valences. In this setting, the mechanisms supporting voluntary visual orientation, under the influence of the emotional components of stimuli, are unknown. We employed a mosaic of pictures with different emotional valences (positive, negative, and neutral) and explored the dynamics of attentional visual orientation, assessed by eye tracking and measurements of pupil diameter. We found that pictures with affective content display increased dwelling times when compared to neutral pictures with a larger effect for negative pictures. The valence, regardless of the arousal levels, was the main factor driving the behavioral modulation of visual orientation. On the other hand, the visual exploration was accompanied by a systematic pupillary response, with the pupil contraction and dilation influenced by the arousal levels, with minor effects driven by the valence. Our results emphasize that arousal and valence should be considered different dimensions of emotional processing both interacting with cognitive processes such as visual attention.

## Introduction

In vision as well as in other sensory modalities, our capacity to process stimuli is limited. Therefore, attention or the ability to select relevant information for the current task is central to adaptive behavior and survival (Raz and Buhle, 2006). One mechanism of attentional capture emphasizes an exogenous process, referring to the automatic drive triggered by highly salient stimuli in a bottom-up fashion. On the other hand, an endogenous process occurs when attention is voluntarily directed to the stimulus in a top-down fashion (Corbetta and Shulman, 2002). The biased-competition model states that it is the interaction of bottom-up afferences entering our visual field with top-down cognitive efferences that gives efficiency to the process of visual attention (Desimone and Ducan, 1995). For both mechanisms, different neural networks have been identified (for a review, see Corbetta et al., 2008). Although these networks act in constant communication, the two of them guide the ocular movements through the superior colliculus (Fecteau, 2004). The fact that the eyes fixate where the attention is located unless a voluntary effort is done to avoid such response makes the ocular behavior a frequent marker of visual attention (Krauzlis et al., 2013).

On visual attention, many efforts were dedicated to establish how the physical properties of the stimuli, such as color, contrast, or orientation, could explain exploratory behavior and how these characteristics fit in saliency models capable of predicting ocular exploration (Itti and Koch, 2001; Parkhurst et al., 2002; Masciocchi et al., 2009; Russell et al., 2014). However, during natural viewing, as physical properties of the stimuli engage automatic bottom-up mechanisms of attentional capture, top-down processes also influence attentional driving, thereby adding complexity to the attentional selection (Borji et al., 2013).

This may explain why saliency models do not perform well in free viewing paradigms, where the effect of voluntary behavior is larger than in restricted detection tasks. The actual trend in saliency modeling is to include cognitive factors in order to increase the accuracy of the models (Navalpakkam and Itti, 2005; Koehler et al., 2014).

One of the many cognitive factors related to visual attention is emotional processing. From an evolutionary perspective, emotions are essential to quickly identify potential threats or benefits and perform appropriate responses to secure survival (Damasio and Carvalho, 2013).

The emotional experience is accompanied by a series of physiological responses, which are triggered in an involuntary and unconscious fashion. The physiological responses are under autonomic control and involve changes in blood pressure, heart frequency, skin conductance, and pupillary diameter. Pupillary diameter reflexively regulates the amount of light that enters the eye but also changes its size when the subject performs a cognitive effort (Granholm and Steinhauer, 2004). A particular enlargement of the pupil has been observed when the subject is exposed to auditory (Partala and Surakka, 2003) or visual emotional stimuli. When subjects are exposed to single pictures, the pupillary response significantly differs between pictures with and without emotional content, independently of the specific hedonic value (Bradley et al., 2008).

Previous studies have explored the relationship of emotional content with visual attentional orienting using ocular behavior (Rösler et al., 2005; van Reekum et al., 2007), microsaccadic rate (Kashihara et al., 2014), or event-related potentials (ERP) (Brosch et al., 2008; Leite et al., 2012) as markers of attention. They found a clear link between emotion and attention when stimuli must be detected or identified. These paradigms explore predominantly the automatic component of attention and usually lack any emotional correlation to give an account of the response triggered within the subject. Even though automatically driven attention is essential to quickly perceive relevant stimuli, most of the time, during natural viewing, multiple stimuli coexist and the subject voluntarily changes the target attended, according to the needs of the current task or goal to accomplish. This voluntary component, which considers the top-down contribution of the emotional processing to the attentional orienting, has been highly neglected (Mohanty and Sussman, 2013).

We conjecture that the emotional content of the visual object would contribute to enhance the saliency of elements of visual scenes and thus contribute to modulate visual exploration toward the emotional images, increasing the time spent on emotional pictures. In agreement with previous evidence, we also expect the magnitude of the pupillary response to be similar when attention is located on emotional stimuli regardless of the hedonic value and to significantly differ when attention is located on non-emotional stimuli. By showing an arrangement of pictures with different emotional content, we examined the attentional bias toward emotional stimuli during voluntary orienting of attention, differentiating the contribution of the emotional valence and arousal.

## Materials and Methods

### Participants

Twenty-eight (14 males) healthy volunteers, ranging from 20 to 37 years (*M* = 28 years, DS 4.9) participated in this study. All participants had normal or corrected to normal vision, did not report any psychiatric or neurological condition, did not consume drugs that acted on the nervous system, and gave written informed consent. The experiment was approved by and was carried out in accordance with the recommendations of the Human Research Ethics Committee from the Facultad de Medicina, Universidad de Chile. All subjects gave written informed consent in accordance with the Declaration of Helsinki.

### Stimuli

We constructed 84 full-color mosaics of pictures from the International Affective Picture System (IAPS) (Lang et al., 1997). Each mosaic contained nine images (sub-pictures) – three of negative (NEG), three of neutral (NEU), and three of positives (POS) emotional value – giving a total amount of 756 different pictures. We designed the stimuli according to the valence and arousal scores of each picture in order to have the least possible variability of arousal level within the emotional categories of each mosaic. We also arrange the mosaics to have one sub-picture of each valence in every row and column, thereby carefully avoiding the adjacency of two sub-pictures of the same valence.

To ensure that images differ from each other, we performed a t-test on the arousal scores [*t*(754) = 41.79; *p* < 0.001] and an ANOVA on the valence scores [*F*(2,753) = 3285.77, *p* < 0.001]. Multicomparison analysis with Bonferroni correction indicated significant differences between all the pairs of valences (positive vs. neutral and positive vs. negatives, *p* < 0.001; negatives vs. neutral, *p* < 0.001).

### Apparatus and Procedure

**FIGURE 1:**
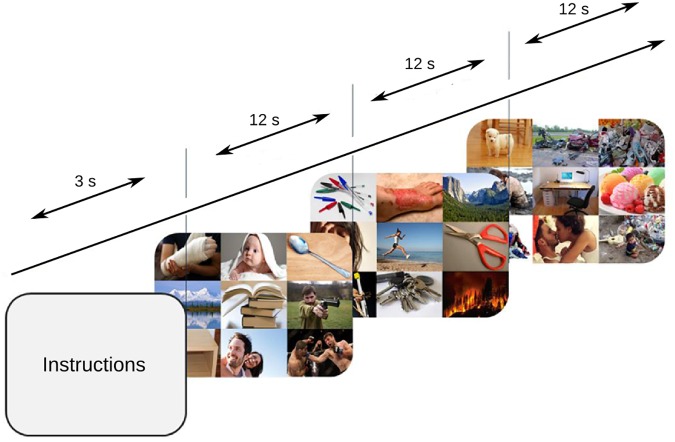
Task design. 84 mosaics were consecutively shown, and every mosaic was presented for 12 s while the subjects freely explored.

The participants were seated in a darkened room, with their head comfortably resting upon a head stabilizer. The stimuli were presented in an LED monitor (ViewSonic, 27^′′^), 52 cm from the subject, covering a visual field of 33° × 49.5°. Three blocks of 27 consecutive trials were displayed. The mosaics were randomly presented for 12 s each – a period during which participants were instructed to free explore (Figure 1) while ocular behavior and pupillary diameter were binocularly recorded with an eye tracking system (Eye Link 1000^®^, SR Research, Ltd., Mississauga, ON, Canada) at a 500 Hz sampling rate. A calibration adjustment was performed before the starting of a new block. An initial screen was presented at the beginning of the trial and no fixation point was included in any of the stimuli.

### Behavioral and Pupillary Data Analysis

The recorded ocular behavior and pupil data were processed and analyzed with custom-made MATLAB scripts. Fixations and saccades are classified by the Eye Link 1000 tracker using the default 30°/s velocity, and 8000°/s^2^ acceleration, for saccade detection. Fixations were used to establish the position of the eyes within each one of the nine picture mosaics. We defined the behavioral variable “dwelling time” as the sum of the time spent on each sub-picture inside a mosaic. We determined the values for each variable according to the emotional category (positive, negative, or neutral), the arousal level (high or low), and the interaction of both (positive-high, positive-low, negative-high, negative-low, and neutral).

The pupillary signal was processed as follows: blinks and offsets were cleaned by linear interpolation and a low-pass filter was applied. Raw data was normalized to the z score in order to control the pupil size variability’s inter-subjects. Transitions were defined as the moments when the eyes abandon one picture (first picture) and enter another one (second picture) within a mosaic. The baseline was calculated as the average signal of the 100 ms before a transition. Because of the temporal pattern of the pupil dynamics, transitions with total times shorter than 200 ms in the first picture and 500 ms in the second picture were not considered.

The average curves for each subject were grouped according to the emotional and/or arousal value of the second picture (any to positive/negative/neutral, any to high/low arousal or any to positive-high/positive-low/negative-high/negative-low). We examined different features of the pupillary signal conforming to the peak values of contraction and dilation after each transition, which included maximum contraction magnitude, the latency of maximum contraction, the speed of contraction, maximum dilation, the delta of dilation, the latency of maximum dilation, and speed of dilation.

For the statistical analysis of the behavioral and pupillary data, we employed distribution testing (Kolmogorov-Smirnov and Shapiro-Wilk tests), group comparisons (Friedman test ANOVA), paired comparisons (Wilcoxon signed rank test), and multiple comparisons with Holm-Bonferroni correction. Classic null hypothesis significance testing and the p-value it delivers depend on the sample size, sample type, type of test, and effect size. Complementary to significance testing, effect size measurements were equally considered. We implemented Cohen’s U3 coefficient, a measure which considers the distribution of one sample in comparison with the median of other samples, indicating the difference between those samples independently of their size (Hentschke and Stüttgen, 2011). 2-n ANOVA was applied to determine the contribution of valence and arousal when both dimensions were considered at the same time.

## Results

### Behavioral Data

**FIGURE 2:**
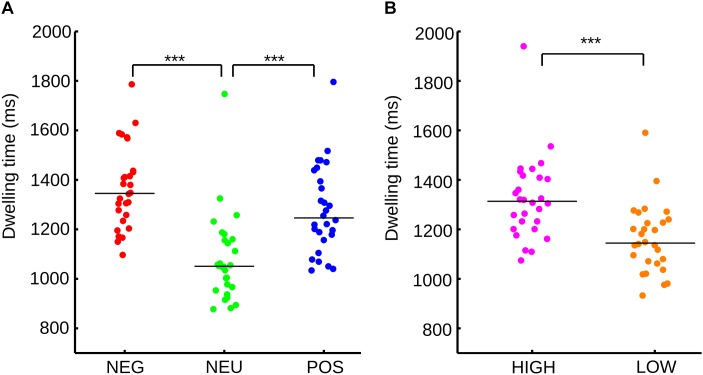
**(A)** Distribution and median of the sample for dwelling time for each emotional category according to valence. **(B)** Distribution and median of the sample for dwelling time for each emotional category according to arousal. ^∗∗∗^*p* < 0.001.

When subjects freely explored a sequence of mosaics of nine pictures, we found significant differences between emotional categories (Friedman test, *p* < 0.001). Sub-pictures with emotional content concentrate larger dwelling times (total time fixated on the images, Figure 2A) than neutral pictures (NEG vs. NEU *p* < 0.001; POS vs. NEU, *p* < 0.001). Negative and positive pictures didn’t show significant differences in the dwelling times (*p* = 0.326). The effect sizes for the dwelling time were calculated using U3 coefficients. U3 values restated the large disparity between emotional categories and the neutral category (NEU vs. NEG, U3 = 0.96; NEU vs. POS, U3 = 0.89) and the smaller difference within the emotional categories (POS vs. NEG, U3 = 0.68).

As each picture has independent valence and arousal scores, the sub-pictures were divided according to only their arousal level. Neutral images were not considered because they have low arousal scores. As expected, high arousal sub-pictures show a significant increase in dwelling time (Figure 2B) in comparison to low arousal pictures (Wilcoxon signed rank, *p* < 0.001). The size effect reinforces the same results (U3 = 0.92).

**FIGURE 3:**
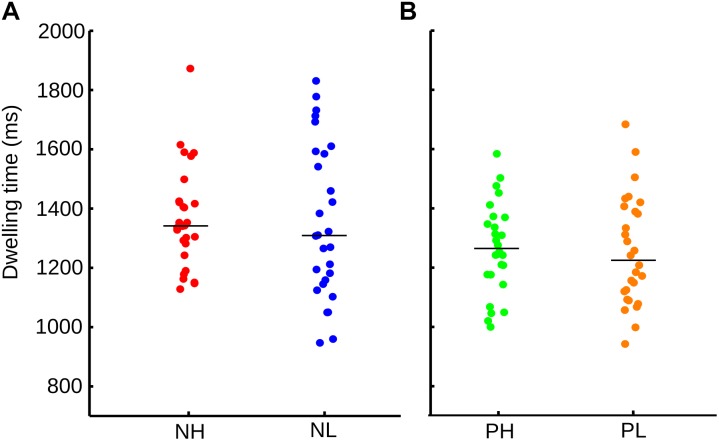
**(A)** Distribution and median of the sample for dwelling time for each emotional category according to valence and arousal. NH, negative-high arousal; NL, negative-low arousal. **(B)** Dwelling time for PH, positive-high arousal; PL, positive-low arousal. Here, images with neutral valence were not included in the calculation.

These results did not clearly dissociate the influence of valence and arousal from the visual orienting of attention; therefore, the data was regrouped. Every emotional category was now divided into low and high arousal and dwelling time once again assessed. These four new categories were negative and high arousal pictures (NH), negative and low arousal pictures (NL), positive and high arousal pictures (PH), and positive and low arousal pictures (PL). We found no significant differences within each emotional category when the arousal level was considered (Figure 3). In Figure 3B, the neutral images were not included. In this figure, we match the number of samples, by randomly selected images form the positive-high-arousal set, to precisely match the number of samples in the PL.

However, an ANOVA test indicated that it was the valence of the picture [*F*(1,108) = 4.71, *p* = 0.032], and not the arousal [*F*(1,108) = 0.38, *p* = 0.541] nor the interaction of them (arousal x valence) [*F*(1,108) = 0.11, *p* = 0.733], explained the differences in dwelling time between conditions. Thus, although the visual exploration differs according to the valence and arousal of the images, the valence of the picture is the main factor driving the observed ocular behavior.

We also examined whether the subjects decrease the exploration of the mosaic images and whether this means a reduction of the number of transitions between images of a mosaic across the trial. We found that subjects systematically decrease their exploration of the mosaics but only decreased up to 30% [Figure 6B, one way ANOVA, *F*(5,162) = 11.4, *p* < 0.001]. This reduction in exploration is consistent with previous observation that demonstrated that subjects initially globally explore the image to then move into more local regions (Ito et al., 2017).

### Pupillary Data

Because the visual voluntary orienting of attention does not require entering consciousness, and there is evidence of an autonomic activation associated with cognitive processing, we wanted to explore whether the pupillary response was modulated by the emotional valence of the stimuli.

**FIGURE 4:**
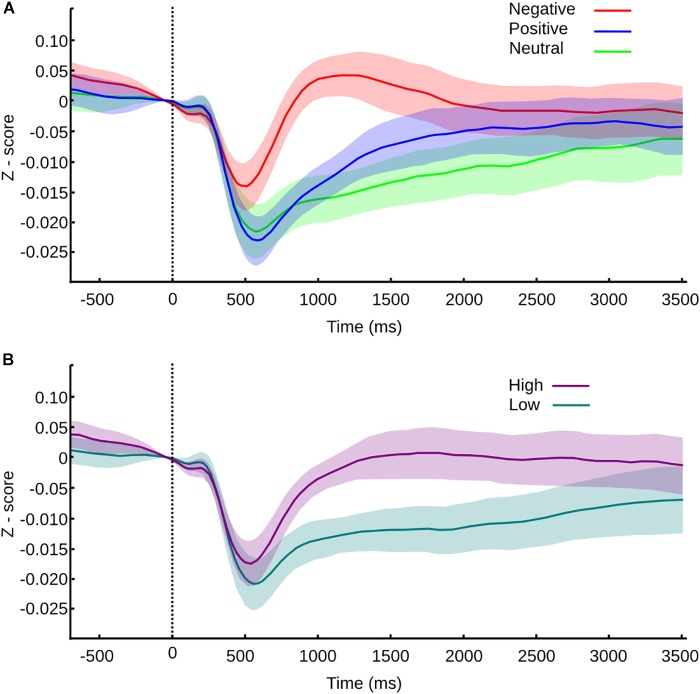
Pupil size values normalized to *z*-score and confidence intervals for transition between pictures within a mosaic. **(A)** Pupil size values for transitions according to valence. **(B)** Pupil size values for transitions according to arousal. Vertical line at time 0 represents the moment of the transition, and the color or the curve represents the category of the picture transitioned to.

Exploring the same valence categorization (positive, neutral, and negative), we found that immediately after the transition of one picture to another, within the same mosaic, the pupil exhibits a fast contraction that reaches its smallest value around 500 ms. This response was followed by a dilation phase, with a maximum value of 1000–2000 ms. Figure 4A shows the normalized average curve of pupillary response according to the valence of the picture the subject transits.

During the contraction phase, all the variables studied showed significant differences (Friedman test, MC *p* < 0.001, SC *p* < 0.001, LC *p* < 0.001). Between categories, neutral and positive picture do not present significant differences in any of the parameters studied – maximum contraction (MC *p* = 1), the latency of maximum contraction (LC *p* = 1) and speed of contraction (SC *p* = 1). Negative pictures trigger the same curve pattern but with smaller contraction, shorter latency, and slower speed of contraction; all of them are statistically significant in comparison to neutral (MC *p* < 0.001, U3 = 0.78; LC *p* < 0.001, U3 = 0.19; SC *p* < 0.001, U3 = 0.85) and positive pictures (*p* < 0.001, U3 = 0.85; LC *p* < 0.001, U3 = 0.15; SC *p* < 0.001, U3 = 0.74). This result suggests that the autonomic response triggered by the pictures is affected by the hedonic value, resulting in a smaller and slower contraction phase for negative pictures.

After the contraction phase, maximum dilation (MD), delta of dilation (DD), and speed of dilation (SD), significantly differ between emotional categories (MD *p* < 0.001, and SD *p* < 0.001; DD *p* = 0.004). The latency of dilation didn’t show differences between categories (LD *p* = 0.1). Negative pictures compared to positive pictures show significant differences only in maximum dilation (MD *p* = 0.0429, U3 = 0.85) and speed of dilation (*p* = 0.029, U3 = 0.78). The lack of difference in the delta dilation (DD *p* = 1) reflects a similar pupil size change, thus we could infer a similar autonomic activation, although the response is faster for negative pictures. Between the negative and neutral pictures, we found significant differences in MD (*p* < 0.001, U3 = 0.96), DD (*p* = 0.013, U3 = 0.85), and SD (*p* < 0.001, U3 = 0.85), giving an account of faster and larger dilation responses for negative pictures. Lastly, when neutral pictures were compared with positive pictures, we found a significant difference only in DD (*p* = 0.013, U3 = 0.85); hence, the pupil reactivity is larger but not faster for positive pictures in comparison with neutral pictures.

As we mentioned before, all pictures have independent valence and arousal scores. The disparities observed in our first analysis could be entangled with the arousal effects. To explore the influence of arousal in the pupillary response, the same way as we did with the behavioral analysis, we divided every transition according to the arousal score of the pictured transitioned to. The average normalized curve of pupillary response according to the arousal level is shown in Figure 4B.

We found that every variable studied was influenced by the level of arousal, where significant differences exist from the contraction to the dilation phase. High arousal pictures triggered contractions of smaller amplitude, which occurred earlier in time and with slower speed when compared to low arousal pictures (Wilcoxon rank test, MC *p* < 0.001, U3 = 0.19; LC *p* = 0.002, U3 = 0.63; SC *p* < 0.001, U3 = 0.3).

During the dilation phase, the pupillary dilation observed was larger in amplitude for high arousal pictures, the DD was also larger, and the peak of amplitude was reached later and slower than the one triggered by low arousal pictures (Wilcoxon rank test, MD *p* < 0.001, U3 = 0.3; DD *p* < 0.001, U3 = 0.074; LD *p* = 0.008, U3 = 0.3; SD *p* = 0.007, U3 = 0.3).

Because the pupil was responsive to the valence and the arousal of the pictures, when they were explored independently, it wasn’t clear which variable was driving the autonomic response. Accordingly, to the same categorization used for the behavioral analysis, we divided every emotional valence depending on its arousal level. Neutral pictures have low arousal scores, so they were excluded from this analysis.

**FIGURE 5:**
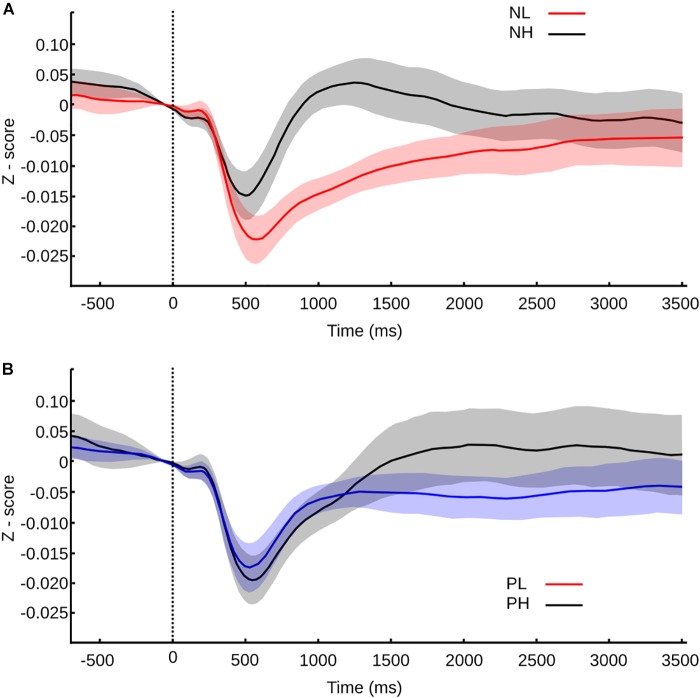
Pupil size values normalized to *z*-score and confidence intervals for transition between pictures within a mosaic according to valence and arousal. Time 0 s represents the moment of the transition, and the color or the curve represents the category of the picture transitioned to. **(A)** Pupil size values for negative pictures according to arousal. **(B)** Pupil size values for positive pictures according to arousal.

The pupillary response to negative pictures showed a clear modulation that began in the contraction phase (Figure 5A). High arousal negative pictures triggered a decrease in the pupillary diameter of significant smaller amplitude, larger latency, and slower speed (Wilcoxon rank test, MC *p* < 0.001, U3 = 0.15; LC *p* < 0.001, U3 = 0.81; SC *p* < 0.001, U3 = 0.3). After the initial contraction, the pupillary diameter dilated in both cases but with significantly larger maximum dilation, the delta of dilation, and faster speed, in the case of high arousal negative pictures (Wilcoxon rank test, MD *p* < 0.001, U3 = 0.04; DD *p* = 0.015, U3 = 0.37; SD *p* < 0.001, U3 = 0.22). Thus, within the negative category, the arousal significantly impacts the pupillary reactivity from contraction to dilation phase.

For positive pictures, the differences were evident only within the dilation phase. During contraction, only the latency of contraction was significantly smaller for the high arousal pictures in comparison to the low arousal pictures (Figure 5A) (Wilcoxon rank test, LC *p* = 0.025, U3 = 0.33). The dilation phase had a similar speed for high and low arousal pictures; however, for positive-high arousal pictures, the amplitude peak was significantly larger and was reached later in comparison to positive-low arousal pictures (Wilcoxon rank test, MD *p* = 0.012, U3 = 0.33; DD *p* < 0.001, U3 = 0.3; LD *p* = 0.018, U3 = 0.3). Then, in the case of positive pictures, the arousal level can also influence the pupillary reactivity, though in the dilation phase mainly and in a milder way than the one found within the negative category.

The ANOVA test indicated that the differences observed during the contraction phase weren’t due to the valence or the arousal independently but due to the interaction of valence and arousal [CM *F*(1,104) = 7.52, *p* = 0.007; LC *F*(1,104) = 10.4, *p* = 0.002; SC *F*(1,104) = 3.9, *p* = 0.0499]. For the dilation phase we found that the maximum and delta of dilation depended on the arousal exclusively [MD *F*(1,104) = 32.2, *p* < 0.001; DD *F*(1,104) = 15.3, *p* < 0.001], while the speed of dilation depended on the arousal [SD *F*(1,104) = 6.28, *p* = 0.014] and also on the interaction between valence and arousal [SD *F*(1,104) = 7.1, *p* = 0.009], and latency of dilation on the interaction [LD *F*(1,104) = 4, *p* = 0.048].

Overall, our data demonstrated that while ocular behavior was explained by the valence of the pictures observed, the pupillary response, which gives us an approximation of the autonomic state linked to the emotional process, was mainly influenced by the arousal level of the pictures with a much smaller effect of the valence.

## Discussion

In our study, we investigated the influence of the emotional component over the visual attentional process. When subjects explored an arrangement of emotional pictures in a free viewing paradigm, we evidenced a facilitation exerted by the emotional component of the pictures over the voluntary visual attentional driving. We described a bias toward hedonic pictures due to the valence and not the arousal dimension of the images. This bias is characterized by an increased time spent observing emotional pictures, especially the ones of a negative nature. At the same time, the pupillary recording gave us an indirect measurement of the autonomic state experience during the gaze shifting, and we determined that although the valence is relevant to the visual behavior and the emotional state triggered, the effect is mainly due to the arousal level of the pictures with some level of interaction with the valence dimension.

The attentional bias to emotional stimuli had previously been studied with different strategies. The first approach investigated how the exposure to an emotion can favor or diminish the performance of subjects during an attentional task like emotion recognition (Brosch et al., 2008) or flicker detection tasks (Bendall and Thompson, 2015). In this paradigm, attention and emotion are treated as separate processes that could help or interfere with each other. A second and one of our interests explore how attention is directed, maintained, or disentangled from stimuli that have inherent emotional value. When subjects observe a single natural image, the pattern of exploration differs between negative and positive pictures (Kaspar et al., 2013). The change observed in the ocular behavior during a free viewing task reflects a top-down influence that could be understood as a way to increase the information extracted from the picture, though the impossibility to avoid the stimulus hampers any chance to extrapolate this observation to an ecological situation.

The top-down modulation is also present from the attentional capture to the maintenance since it influences the saccades programming (Nummenmaa et al., 2009) and the dwelling time on the image (Rösler et al., 2005). Not many studies have compared natural images simultaneously presented, but the scarce evidence uses only two pictures with a different pairing of neutral and affective images and with dissimilar results. In one case, neutral pictures were contrasted to affective pictures and it was concluded that a similar emotional bias exists toward the negative ones (Rösler et al., 2005). Recently, another study explored the ocular behavior when positive and negative images were suddenly and simultaneously presented, thereby describing an emotional bias toward positive pictures (Fernández-martín and Calvo, 2016).

Single presentation of stimuli or simultaneous and sudden presentation of two or more pictures do not necessarily explain the process of visual orienting of attention in an ecological situation, where the emotional context is neither homogeneous nor changes randomly and constantly. With our paradigm, we approached the behavior in a better way in an open uncontrolled situation. By allowing the subjects to explore self-limited pictures of opposite emotional value for a long period of time and in a free fashion, they had the chance to look for or avoid the perception of any emotion.

To distinguish the stages of attention within a task, some authors have used the terms vigilance and maintenance referring to the initial detection of and the posterior dwelling on a stimulus accordingly (Armstrong and Olatunji, 2012). Using the same terminology, our paradigm did not explore the vigilance since a complex and heterogeneous arrangement of pictures was always present, but it allowed us to explore the maintenance and disengagement of attentional resources when subjects swiftly and constantly shifted from picture to picture within a mosaic. The fact that emotional pictures are observed for longer periods of time could be seen as an increase in the time dedicated to affective images or as the consequence of the lack of relevance of neutral images. Neutral pictures usually contain inanimate objects and have simpler compositions than affective pictures. The presence of animated objects automatically allows the subject to easily confer a more complex semantic meaning, nonetheless the significant difference observed between negative and positive pictures couldn’t be explained by the semantic complexity of the picture, given the fact that negative and positive pictures have many elements and similar elaboration.

**FIGURE 6:**
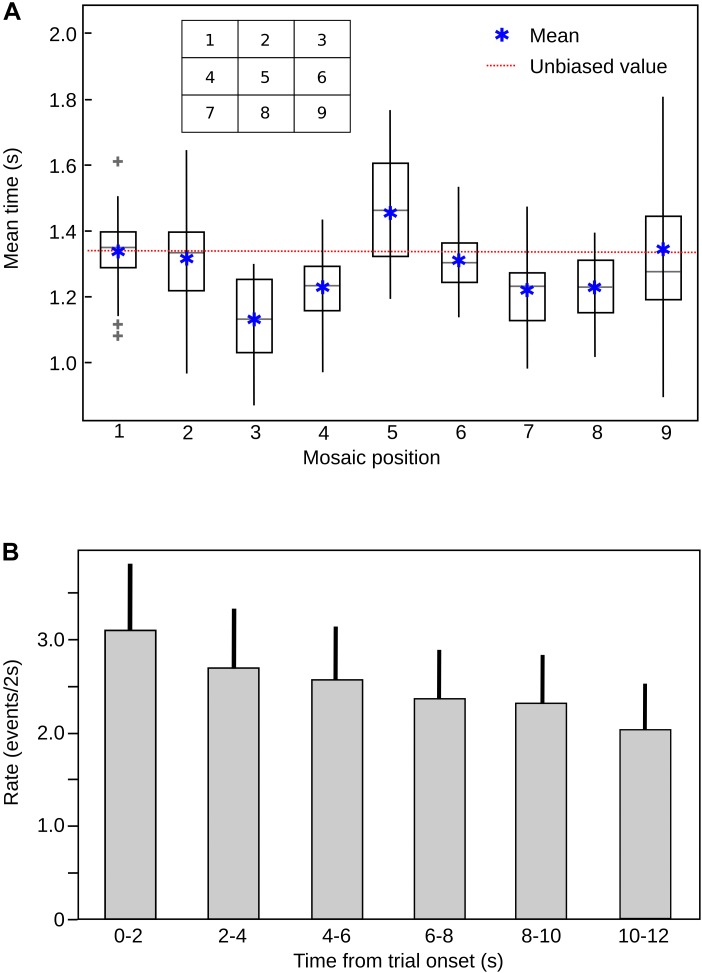
**(A)** Mean fixation time in each quadrant (mosaic position), across subjects. The most frequent fixation quadrant corresponds to the central bias, demonstrating the classical central bias for image fixation. The box represents the 25% and the lines the 75%, of the data distribution, respectively. The gray line inside the boxes represent the median of the data and the gray plus signs the outliers. The red line depicts the values corresponding to the unbiased (random) distribution (11, 11%). **(B)** Rate of image transitions in each mosaic stimulus, across trial time. The rate of exploration decreases significantly with time. ANOVA, *F* = 11.4, *P* = 1.72e-09. Bars are mean values for each 2 s epoch and the lines above the bars represent the SD.

It is possible that along with an emotional bias, other spatial biases are present. The central fixation bias indicates that the central area of a picture always concentrates a larger amount of fixations in comparison to the periphery of the image (Tatler, 2007). In our arrangement, the central fixation bias was present, but we did not observe a significant bias for the upper-left or right-inferior corners that could be explained by a similar exploration logic during scene exploration and reading (Luke and Henderson, 2016). These biases can be seen in the meant fixation time spend in each of the nine quadrants (Figure 6). We carefully considered those spatial biases during the design of the mosaics, so there is an equal number of pictures of each valence in every position within the arrangement; thus our results reflect only the influence of the affective meaning and not any other confounding variable.

Our results are in the same line of other authors who have established an independent mechanism for processing arousal and valence dimension of emotional stimuli. Although many studies explore the cerebral activity when the valence or the arousal vary (Nielen et al., 2009; Ni et al., 2011; Wiens and Syrjänen, 2013; Mathieu et al., 2014; Sieger et al., 2015), the impact of each dimension on the ocular behavior was neglected. All the evidence available refers to the valence of the images as the main emotional characteristic that drives the visual exploration (Rösler et al., 2005; van Reekum et al., 2007; Kaspar and König, 2012).

When we separated our data according to valence and arousal levels, we observed that the main variable that influences the ocular behavior is valence, but arousal does have an impact on the emotional state of the subject experiments. We considered it essential to include a metric as the pupillary diameter to give an account of the emotional state triggered by the pictures. All the studies previously mentioned that relate emotion to visual attention rely on the preconceived hedonic value of the pictures, lacking any metric indicating the adequate perception of the picture and if the behavioral variables explored are accompanied by some physiological specific response.

Previous work using single emotional pictures had described how the pupil was modulated by the emotional value of the stimuli in dissimilar situations. One study established that when subjects listened to emotional sounds, they experiment an increase in their pupillary diameter for negative and positive emotions (Partala and Surakka, 2003). The modulation seen was context related, suggesting a degree of regulation by the attentional processes (Steiner and Barry, 2011). In the visual modality, the evidence suggests the existence of a regulation from the initial contraction after the presentation of a stimulus (Henderson et al., 2014) to the consecutive dilation phase (Bradley et al., 2008; Kashihara et al., 2014). A common practice in these studies is the use of the scale of millimeters to assess the pupillary changes – a metric that we discarded in favor of a normalized value since the absolute diameter changes between subjects.

One interesting aspect of the pupil response in our parading, is that although the temporal profile of the pupil response characteristically extend over 1 or 2 s (Bradley et al., 2008), our pupil data shows the same pattern of response, despite transition to other images within the mosaic. This implies that 500 ms of viewing into a picture is long enough to trigger the classic contraction-dilation curve even if the subject shifts its gaze constantly to another image. A second aspect is that previous studies considered only the valence of the pictures. This is not a minor detail because we found that the modulation observed for affective pictures, especially the negatives, is in fact an effect of the arousal of the pictures. Although it is true that our analysis shows some effect of the valence on the contraction phase for the positive pictures and in the contraction and dilation phase for negative pictures, these regulations depend on the existence of an arousal variation. The greater differences found for the negative pictures in comparison to the positive pictures are probably due to the dissimilar level of arousal the images have. When we designed the mosaics, the arousal level was controlled to have a mean value as similar as possible to the emotional categories; however, since the number of pictures that suited the study was limited, negative pictures still had a higher arousal mean. In conclusion, the emotional value of the stimuli modulates the visual attentional orienting, modifying the time the subjects spend exploring the affective pictures so the negative stimuli are observed for longer times than the positive stimuli. At the same time, the emotional nature of the pictures is quickly recognized, triggering an autonomic response of which magnitude and temporal profile depends mainly on the arousal dimension of the stimuli.

Our study supports the idea of differential processing of valence and arousal and provides a novel paradigm which allows the study of the voluntary orienting of visual attention and delivers an accurate metric of pupillary response that could be used in ecological situations.

## Author Contributions

CA and PM designed the study and wrote the manuscript. CA and KM carried out the data acquisition and analyzed the data.

## Conflict of Interest Statement

The authors declare that the research was conducted in the absence of any commercial or financial relationships that could be construed as a potential conflict of interest.
